# Biometric and refractive changes following the monocular application of peripheral myopic defocus using a novel augmented-reality optical system in adults

**DOI:** 10.1038/s41598-022-15456-4

**Published:** 2022-07-13

**Authors:** Ryo Kubota, Nabin R. Joshi, Tara J. Fitzgerald, Inna Samandarova, Maksud Oliva, Arkady Selenow, Amitava Gupta, Steven Ali, G. Lynn Mitchell, Robert Chun, Kenneth J. Ciuffreda

**Affiliations:** 1 Kubota Vision Inc., Seattle, WA USA; 2 Kubota Pharmaceutical Holdings Co., Ltd., Tokyo, Japan; 3 Manhattan Vision Associates – Institute for Vision Research, New York, NY USA; 4 The Ohio State University College of Optometry, Columbus, OH USA; 5 State University of New York (SUNY) College of Optometry, New York, NY USA

**Keywords:** Diseases, Medical research

## Abstract

The prevalence of myopia is growing at an alarming rate and is associated with axial elongation of the eye. The cause of this undesirable physiological change involves multiple factors. When the magnitude of myopia approaches high levels, this accompanying mechanical effect increases the risk of developing other clinical conditions associated with permanent vision loss. Prior work has investigated how we may halt or reverse this process of axial elongation associated with myopic progression when we expose the eye to a peripheral myopic defocus stimulus. Specifically, the known, short-term response to myopic defocus stimulation is promising and demonstrates the possibility of establishing more permanent effects by regulating the axial length of the eye with specific defocus stimulation. However, how to directly convert these known, short-term effects into more long-term, permanent changes to effectively prevent these unfavourable physiological and refractive changes over time is yet to be understood. Here, we show for the first time that we can produce sustained, long-term reductions in axial length and refractive endpoints with cumulative short-term exposure to specific myopic defocus stimuli using a novel optical design that incorporates an augmented reality optical system. We believe that this technology will have the potential to improve the quality of vision in mankind.

## Introduction

By 2050, 4.8 billion people, or nearly half of the world’s estimated population, will be nearsighted^1^. Myopia, more commonly known as nearsightedness, may develop either gradually or relatively rapidly. In most cases, it can be corrected optically with spectacles, contact lenses, or refractive surgery^2^. It becomes concerning when either the magnitude of myopia or the rate of myopic progression reaches high levels (e.g., greater than −6.00 D spherical equivalent)^3^ since this is associated with the axial elongation of the eye. Thus, myopia increases the risk of not only retinal detachments^4^ but other potentially blinding conditions such as open-angle glaucoma^5^, cataracts^6^, and myopic macular degeneration^3,7,8^. For example, there is a 67% increased risk for developing myopic maculopathy with every diopter increase in myopia^9^. In addition, slowing myopia by 1 diopter (D) will reduce the likelihood of a person developing myopic maculopathy by 40%^9^. Because of these associated risks and increasing prevalence worldwide^1^, myopia and its progression have become a significant public health concern^1,10^ and warrants more advances to target better preventative treatments^2^.

Previous studies have investigated the role of peripheral retinal defocus in myopic progression. There are two main types of spherical defocus: (1) hyperopic defocus, where rays of light focus behind the retina, and (2) myopic defocus, where rays of light focus in front of the retina. It has been shown that peripheral *hyperopic* defocus on the retina is the chief driver of axial elongation of the eye^11–13^, an undesirable trigger for myopic progression. The opposite, or more desirable clinical effect of inhibiting axial elongation, has been demonstrated using peripheral *myopic* defocus in both human^14^ and animal studies^15,16^. These relationships are critical to understand in order to unlock protective pathways to inhibit myopiagenic changes and their potentially adverse effects.

**Figure 1:**
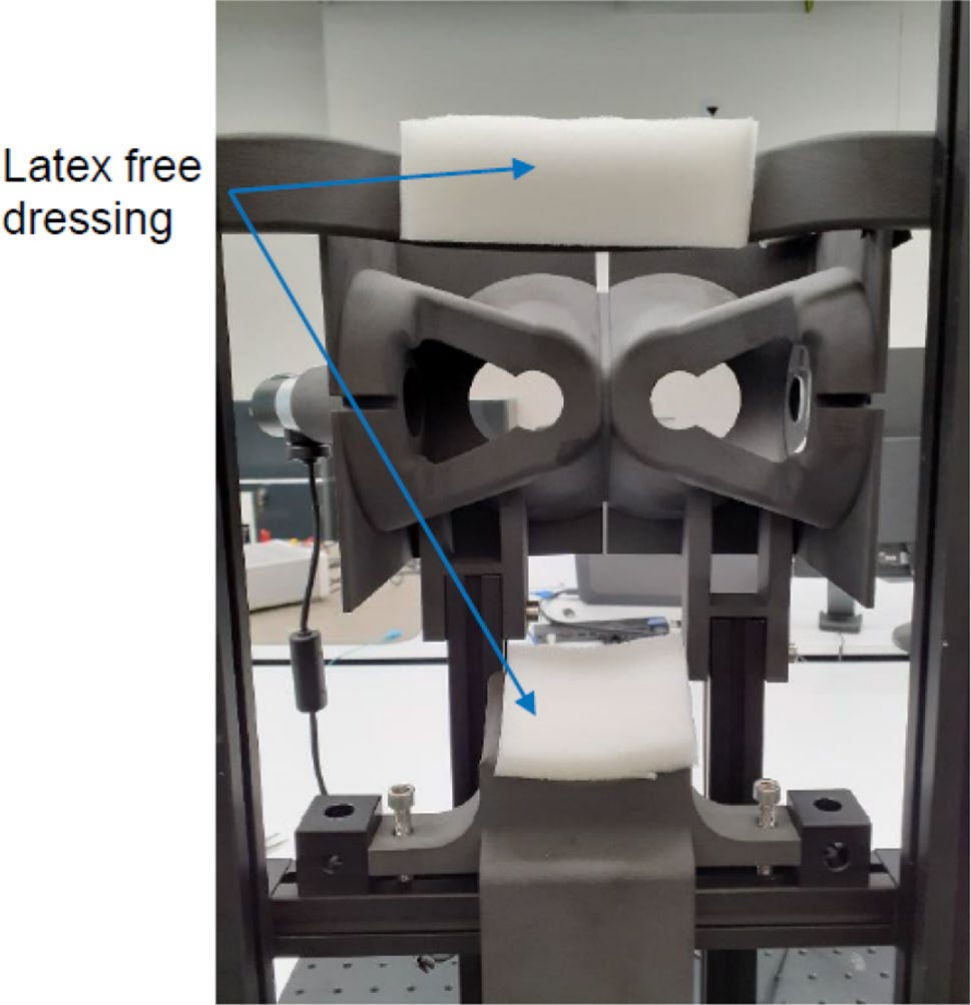
A non-wearable augmented reality (ANWAR) optical system.

**Figure 2:**
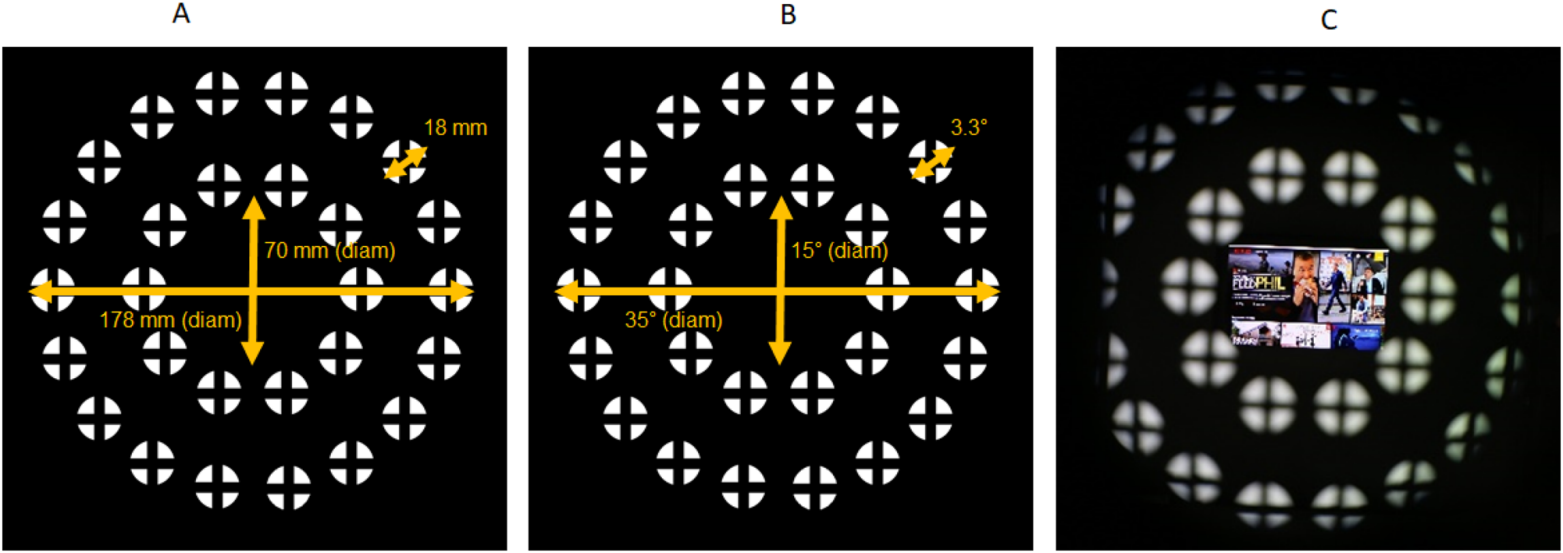
The extent of the stimulus in millimeters (**A**) and degrees (**B**). The image on the far right (**C**) shows the “approximate" subject’s view through the test eye through the ANWAR setup.

The two types of defocus, myopic and hyperopic, can elicit *short-term* (*i.e.,* transient) or *long-term* (*i.e.,* more permanent) physiological responses in the eye. Our preliminary work demonstrated that inhibiting axial length using Fresnel lenses of + 3.50D and + 5.00D to achieve *short-term* peripheral myopic defocus is possible in young adults^17^. Similar work has also established the *short-term* and *long-term*^14,15,17–19^ physiological responses seen in axial length following myopic and hyperopic defocus stimuli in animal^15^ and human studies^14,19^. For example, one study found that human eyes significantly shortened (−8 ± 9 µm) after being exposed to 40 min of *myopic defocus,* with a rapid recovery back to baseline after approximately 35 min^20^. These findings advance our understanding of the temporal dynamics underlying myopic progression. More importantly, we have yet to fully characterise the physiological response between axial length and refractive endpoints to *repeated* myopic defocus stimuli over extended periods. Specifically, we lack an understanding as it pertains to (1) describing the cumulative effect of repeated exposure to myopic defocus stimuli on physiologic measures and (2) identifying how to convert these favourable *short*-term changes into permanent *long*-term changes. Thus, in the present study, we aimed to address whether transient *short*-term changes in axial length and cycloplegic refractive endpoints would lead to sustained *long*-term changes after recurrent weekly exposure (7.5 h/week) for 4 months in young, myopic adults. We accomplished this with a novel optical system using an augmented-reality-like stimulus^21^. The apparatus and the stimulus are shown in Fig. 1 and 2.

## Results

Table 1 presents the demographics of the subjects. There was a large range of refractive errors in the sample extending from −9.76 D to −0.87 D spherical equivalent (SPHEQ). Only one subject presented with a cylinder value greater than one DC. The inter-ocular difference in SPHEQ at baseline ranged from -2.65 D (Subject #6) to -0.070 D (Subject #1). Baseline axial length measurements differed by as much as 754.2 µm, with an average of 235.8 µm. The right eye was longer than the left eye in five of the seven subjects. All but subject #2 had documented worsening myopia of at least 0.50 D (progression in the last year).

**Table 1:** Demographic characteristics and pre-treatment cycloplegic refraction using ANWAR optical apparatus and axial length for enrolled subjects (n = 7).

ID #	Age (yrs)	Gender	Right eye				Left eye			
			Sphere	Cyl	SPHEQ	AxL (µm)	Sphere	Cyl	SPHEQ	AxL (µm)
1	24	F	−1.33	−0.69	−1.68	24,308.3	−1.54	−0.13	−1.61	24,155.0
2	21	M	−1.62	−0.42	−1.83	26,430.0	−1.39	−0.38	−1.58	26,488.3
3	29	F	−0.26	−1.21	−0.87	23,370.0	−0.66	−0.98	−1.15	23,447.5
4	32	F	−0.81	−0.23	−0.92	23,470.8	−0.67	−0.31	−0.82	23,365.8
5	24	F	−5.06	−0.61	−5.37	26,219.2	−3.53	−0.57	−3.81	25,465.0
6	28	F	−9.47	−0.57	−9.76	26,470.8	−6.84	−0.55	−7.11	25,720.8
7	22	M	−4.71	−0.77	−5.09	26,118.3	−4.23	−0.44	−4.45	26,094.2

**Figure 3:**
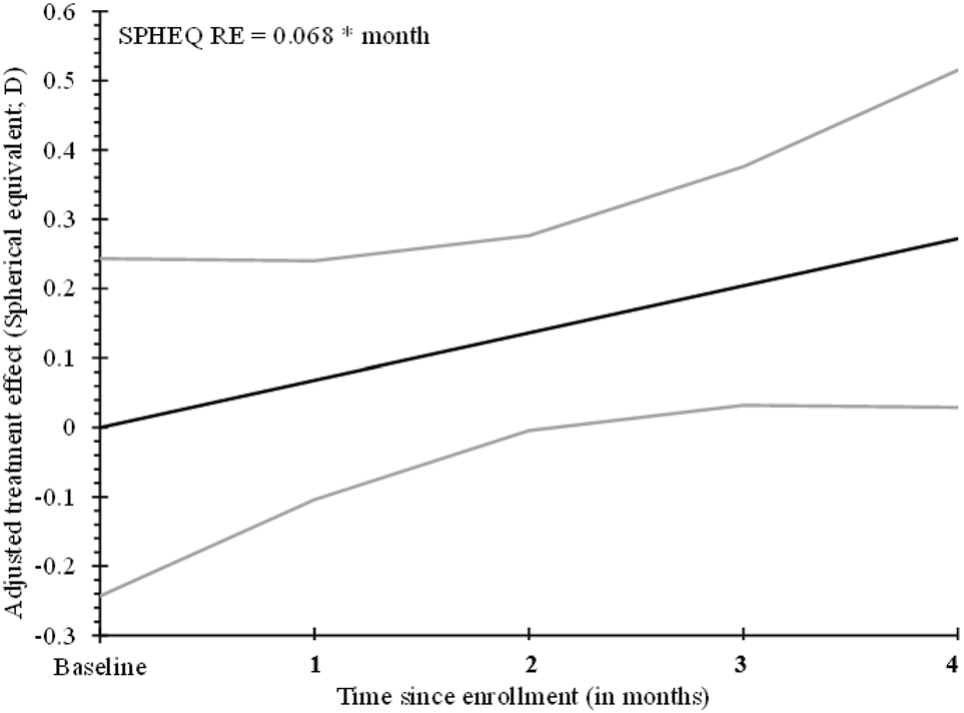
Cumulative adjusted treatment effect based on spherical equivalent refractive error (**D**) using WAM. Light grey lines represent the 95% confidence band around the regression line.

**Figure 4:**
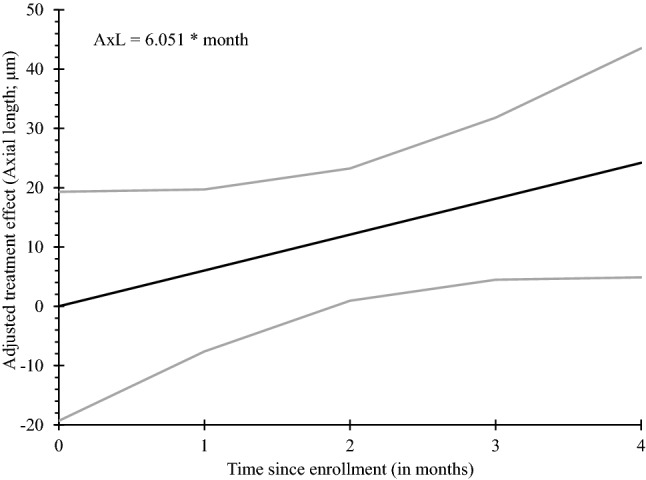
Cumulative adjusted treatment effect based on axial length (microns). Light grey lines represent the 95% confidence band around the regression line.

As shown in Table 2, there was a positive relationship between cumulative effect and study visit for four of the seven study subjects (increasing cumulative adjusted treatment effect from months 1 to 4). This would imply that continued treatment resulted in a reduction in the level of myopia in the test eye for these subjects (Figs. 3, 4).

**Table 2:** Spherical equivalent refractive error at each study visit (using WAM; in diopters).

Visit	Measure	Participant ID						
		1	2	3	4	5	6	7
Baseline	Between-eye difference^a^	−0.07	−0.25	0.28	−0.096	−1.56	−2.65	−0.64
Month 1	Between-eye difference^a^	−0.12	−0.33	0.24	0.43	−1.57	−2.51	−0.63
	Adjusted treatment effect^b^	−0.05	−0.077	−0.047	0.52	−0.013	0.14	0.006
	*Cumulative adjusted treatment effect*	−0.05	−0.077	−0.047	0.52	−0.013	0.14	0.006
Month 2	Between-eye difference^a^	−0.153	0.022	0.82	0.060	−1.71	−2.85	−0.59
	Adjusted treatment effect^b^	−0.083	0.28	0.53	0.16	−0.15	−0.20	0.050
	*Cumulative adjusted treatment effect*	−0.133	0.20	0.49	0.68	−0.16	−0.064	0.056
Month 3	Between-eye difference^a^	0.048	−0.10	1.00	0.097	−1.95	−2.82	−0.42
	Adjusted treatment effect^b^	0.12	0.15	0.72	0.19	−0.40	−0.18	0.22
	*Cumulative adjusted treatment effect*	−0.015	0.35	1.20	0.87	−0.56	−0.24	0.27
Month 4	Between-eye difference^a^	−0.11	−0.37	0.22	−0.14	−1.74	−2.54	−0.70
	Adjusted treatment effect^b^	−0.039	−0.12	−0.059	−0.045	−0.18	0.11	−0.056
	*Cumulative adjusted treatment effect*	−0.054	0.24	1.14	0.83	−0.74	−0.13	0.22

^a^Calculated as treated eye minus control eye. Values greater than zero indicate less myopic eyes.

^b^Calculated as between-eye difference at visit minus the between-eye difference at baseline. Values greater than zero indicate a positive treatment effect (greater positive myopic shift in the treated eye as opposed to control eye).

Using a repeated-measures regression model, the estimated treatment effect improved by 0.068 D (95% CI: 0.011 to 0.125; *p* = 0.011) per month of treatment (Fig. 3); that is, with each month of treatment, the test eye becomes 0.068 D less myopic than the control eye. Given the high variability associated with biological data, model R^2^ was acceptable at 14.9%. Using this estimated slope, the predicted treatment effect after 12 months would be 0.816 D with 95% confidence that the true effect falls within the interval of 0.132 D to 1.5 D. These calculations assume the same pattern of improvement from months 5 to 12, as observed in months 1 to 4.

As was true for the spherical equivalent refractive error, there was a positive relationship between cumulative adjusted treatment effect and study visit for four of the seven study subjects with respect to axial length (Table 3). This implies that continued treatment reduced axial length growth in the test eye for these subjects.

**Table 3:** Axial length at each study visit (in microns).

Visit	Measure	Participant ID						
		1	2	3	4	5	6	7
Baseline	Between-eye difference^a^	153.3	−58.3	−77.5	105.0	754.2	750.0	24.2
Month 1	Between-eye difference^a^	159.2	−45.8	−60.8	101.7	756.7	757.5	−2.5
	Adjusted treatment effect^b^	−5.9	−12.5	−16.7	3.3	−2.5	−7.5	26.7
	*Cumulative adjusted treatment effect*	−5.9	−12.5	−16.7	3.3	−2.5	−7.5	26.7
Month 2	Between-eye difference^a^	140.0	−48.3	−62.5	110.8	735.8	727.5	9.2
	Adjusted treatment effect^b^	13.3	−10.0	−15.0	−5.8	18.4	22.5	15.0
	*Cumulative adjusted treatment effect*	7.4	−22.5	−31.7	−2.5	15.9	15.0	41.7
Month 3	Between-eye difference^a^	125.0	−54.2	−80.0	106.7	759.2	713.3	−7.5
	Adjusted treatment effect^b^	28.3	−4.1	2.5	−1.7	−5.0	36.7	31.7
	*Cumulative adjusted treatment effect*	35.7	−26.6	−29.2	−4.2	10.9	51.7	73.4
Month 4	Between-eye difference^a^	122.5	−56.7	−86.7	110.0	747.5	723.3	−23.3
	Adjusted treatment effect^b^	30.8	−1.6	9.2	−5.0	6.7	26.7	47.5
	*Cumulative adjusted treatment effect*	66.5	−28.2	−20.0	−9.2	17.6	78.4	120.9

^a^Calculated as treated eye minus control eye.

^b^Calculated as between-eye difference at baseline minus the between-eye difference on visit. Values greater than zero indicate a positive treatment effect.

Using a repeated-measures regression model, the estimated treatment effect improved by 6.051 microns (95% CI: 1.500 to 10.604 microns; *p* = 0.006) per month of treatment (Fig. 4); that is, with each month of treatment, the test eye becomes 6.051 microns shorter than that of the control eye. Using this estimated slope, the predicted treatment effect after 12 months would be 72.606 microns with 95% confidence that the true effect falls within the interval of 18.0 to 127.25 microns. These calculations assume the same pattern of improvement from months 5 to 12, as observed in months 1 to 4. Cycloplegic refraction at the end of treatment was 0.055D lower (less myopic) than at the baseline visit (std = 0.088; *p* = 0.16). This corresponded to a 16.33 µm shorter mean eye length after treatment (std = 19.19; *p* = 0.078). Neither change was statistically significant.

## Discussion

The human eye can make compensatory changes in both axial length and refractive state within minutes^19,20^ following brief exposure to a peripherally defocused retinal stimulus. This physiological (axial length) and refractive response is thought to be initiated by the choroid^14–18^. The results of our study extend the earlier work of Delshad et al.^20^, which demonstrated how transient, short-term changes in refractive endpoints and axial length measures in humans respond to short-term (60 min) defocus stimulation. Furthermore, Delshad et al. showed that after eliminating the myopic defocus stimulus in the periphery, the axial length returned to baseline after only 35 min.

In the present study, we extended the aforementioned important findings by showing the sustained, cumulative effects of short-term myopic defocus, resulting in progressive and relatively long-term (over 4-month’s time) effects in axial length and refractive error. We demonstrated that it is possible to achieve a sustainable, physiological effect with regard to axial length measures and related reduction in the magnitude of myopic refractive error following exposure of only 1.5 h of peripheral myopic defocus applied 3–5 times a week over 4 months (7.5 h total defocus time on average per week). The stimuli dimensions are shown in Fig. 2.

When we extrapolate this positive finding over an additional 8 months (i.e., 1 year total), the predicted total change produces an impressive effect of 0.816 D and 72.606 µm in refractive and axial length change, respectively. Since a 5 µm change in axial length is approximately equal to a 0.012 D change in refraction^22^, our new findings represent a significant treatment effect with respect to each endpoint, namely axial length and myopic refractive status.

Our investigation included a novel stimulus and optical design^21^. For the myopic defocus stimulus, a + 3.50 D magnitude was chosen as a result of a previous pilot study using peripherally defocused Fresnel lenses. The current study results demonstrated that this same stimulus achieved a significant and lasting effect after an average of only 7.5 h per week of peripheral stimulation. The luminance of our stimulus was chosen to mimic typical photopic lighting conditions since exposure to higher illumination of the outdoor environment is believed to be one of the contributors to reducing myopia progression in children^23–25^. Furthermore, our novel optical system can be programmed to control the amount of defocus, size, contrast, retinal location, luminance, and chromaticity of the stimulus, which will be investigated in future studies.

Our preliminary findings have several potential implications. They demonstrated the sustained, cumulative, and positive effects of short-term exposure to peripheral myopic defocus. In this study, we developed a novel and revolutionary optical design to treat myopic progression using short periods of exposure to peripheral myopic defocus. Currently, there are treatment options for myopia progression in the form of either dual-focus/bifocal contact lenses^26–28^ or spectacles^29^. However, these options must be used for at least 6–10 h every day to achieve a therapeutic effect of reducing myopic refractive error depending on the method. For example, MiSight contact lenses for myopia control showed a therapeutic benefit at 1 year (0.4 D reduction in myopia compared to the control) when using the lenses for at least 10 h a day for 6 days a week^26,27^. Our preliminary positive findings demonstrate that we may incorporate this innovative design in the near future into a comfortable, wearable spectacle form to achieve a lasting desirable effect using shorter treatment times than other commercially available options—ultimately, to reduce myopia progression in the natural environment of children or adults.

We limited our investigation to adults only and used a single magnitude of defocus (+ 3.50D) and a fixed luminance without choroidal measurements. Future studies will include larger cohorts of children with different levels of defocus and other stimulus parameters to help us determine the most effective stimulus for controlling eye growth, along with choroidal assessment. Also, additional work is needed to advance our understanding of parameter optimisation to maximise efficacy for shortening the axial length and reducing the resultant myopic refractive error, with the goal of attaining permanent effects. We also recognise that shorter daily treatment sessions in conjunction with higher measurement frequency may help define the most efficacious course of treatment.

In summary, our study showed for the *first* time that a reduction in axial length and cycloplegic refraction can produce sustained, *long*-term changes with cumulative exposure to short-term myopic defocus stimulation. These findings can successfully combat the myopic epidemic, which is a worldwide public health problem.

Hours of defocus treatment ranged from 112 to 160 h, with a mean of 150 h (SD = 16.9) over the four months. All participants completed their five study visits.

## Methods

This was a 4-month *prospective* clinical study to investigate the effects of using projected peripheral myopic defocus in a benchtop optical system. The primary objective of our study was to assess central axial length and cycloplegic refractive changes using our novel optical system and myopic defocus stimulus design as shown in Fig. 2. Monocular stimulation was used to compare the effects of peripheral myopic defocus in the test eye (right eye) to no stimulation in the control eye (left eye). Thus, subjects were exposed to the peripheral myopic defocus stimulus for 1.5 h in the test eye compared to the control eye, applied on an average of 5 times a week over 4 months. We hypothesised that we would observe a significant reduction in both the central axial length and cycloplegic refractive endpoint in the test eye compared to the control eye following termination of the projected peripheral defocus sessions.

### Subjects

Seven myopic (spherical equivalent ≥ −0.75 D in each eye) adults aged 18 to 35 years (5 females, 2 males, 25.7 ± 4.0 years) were recruited from a private practice setting in New York, NY (Manhattan Vision Associates) and completed the study. Prior to enrollment, a clinical vision screening was completed to confirm normal visual acuity, normal binocular vision, and the absence of any ocular diseases that would adversely affect visual function. In addition, subjective refraction was completed as part of the visual screening to determine the refractive error of the subjects (mean spherical equivalent = −2.50 D) and confirm that visual acuity was correctable to 20/20 in each eye.

Subjects with anisometropia > 3.00 D, astigmatism > 2.00 DC, or recent history of other myopia control treatments^30^ (e.g., orthokeratology^31^, atropine^32^, bifocal contacts^33^, multifocal spectacle lenses^34^) were excluded from the study. Written informed consent was obtained from all participants prior to enrollment. All study procedures were approved by an independent institutional review board (Sterling Institutional Review Board, Atlanta, Georgia, USA, Study ID 8277) and adhered to the tenets of the Declaration of Helsinki.

### Description of optical apparatus used in the study

For our optical design, we constructed a non-wearable, augmented reality (ANWAR) benchtop apparatus comprised of two partially reflecting mirrors for both eyes, an achromatic lens (aberration minimising lens), a viewing aperture, a chinrest, and a headrest. The overall design was engineered to deliver precise optical vergence. The system allowed each subject to rest their chin and forehead comfortably in the apparatus while looking through a viewing aperture at a distance target 4 m away.

The stimulus for the peripheral defocus was produced by the following sequence: (1) a myopic defocus stimulus image was projected onto the aberration-minimising, achromatic lens, (2) the stimulus image was refracted by the achromatic lens, and (3) the stimulus image was reflected by a mirror onto the peripheral retina.

The myopic defocus stimulus consisted of 28 circular images arranged into two concentric rings. The outer ring consisted of 18 circles, and the inner ring consisted of 10 circles. The diameter of the outer ring was approximately 35°, and the diameter of the inner ring was 15°. Each circular image was designed to have a central cross-hair with a diameter of 3.3°. The stimulus image was derived from an LCD light source.

When seated in the ANWAR apparatus, 15° of the subject’s central field was clear and unimpeded. Thus, each subject could view a central target (a television screen) at a distance of 4 m while being exposed to the surrounding myopic defocus stimuli (as described above) in the periphery. Subjects were fully corrected with custom-made spectacles using their most recent subjective refraction completed at their initial vision screening while seated in the optical apparatus.

The two mirrors reflected the image from the television screen equally in each eye. In addition, there was a uniformly illuminated grey poster (10 lx) surrounding the television monitor screen, which served as a neutral background and filled each subject’s remaining visual field while in primary gaze.

Figure 1 shows the stimulus projected onto the periphery using this bi-ocular setup. The optical apparatus allowed us to control the luminance for each of the three field elements: projected defocus, television screen, and grey poster background. We chose the test luminance condition of the projected defocus stimulus to be 20 times greater than the luminance of the grey poster background (10 lx) based on positive results from an earlier pilot study.

### Study protocol

Once a subject was screened and accepted for enrollment, baseline testing (cycloplegic refraction and axial length) was performed over two visits before starting any defocus sessions. Visual acuity, colour vision, contrast sensitivity, and binocular vision testing (negative/positive relative accommodation, Von Graefe phorias, stereopsis) were also completed at the beginning and at the end of the study to ensure that no changes were detected in the subjects’ overall visual function during the study.

Once all baseline testing was completed, subjects were scheduled to start their defocus sessions (1.5 h of defocus treatment on an average of 5 times a week). Since previous studies have shown axial length to be greatest during the morning hours following a fluctuating, diurnal (circadian) rhythm^35^, all defocus sessions were scheduled to commence between 7:30 AM and 9:30 AM. During these sessions, no other testing was completed, aside from the subject sitting within the ANWAR apparatus and completing their uscheduled defocusing for 1.5 h. Each session was set up so that subjects would defocus for 45 min, take a 5-min rest period, and then complete another period of defocus for 45 min. All subjects completed approximately five visits a week, totalling 20 visits in 4 weeks, 40 visits in 8 weeks, 60 visits in 12 weeks, and 80 visits in 16 weeks. Thus, each subject completed, on average, a total of 7.5 h of myopic defocus each week through the 4-month timeframe.

Subject activity and physical movements were minimised and controlled as much as possible during the scheduled defocus sessions. The left eye served as the control with no exposure to the peripheral defocus stimulus, while the projected peripheral defocus was only applied to the right eye. The ambient light levels were maintained at a photopic level of approximately 230 lx during the testing period. The subjects were seated comfortably and instructed to watch a video in colour on a television monitor (34-inch horizontally by 19-inch vertically) positioned 4 m away through the ANWAR system.

Subjects were evaluated monthly (“non-defocus” visits) during the 4-month study where axial length and cycloplegic refractive measures were obtained. These monthly visits (4 total) lasted approximately 2.5 h.

### Instrumentation for axial length and refractive measurements

The Lenstar LS-900 (Haag-Streit, Mason, OH) was used to measure the axial length. This instrument has a resolution of 0.01 mm and a range of 14–32 mm. First, the subject's head was placed within the forehead/chinrest assembly, with the non-tested eye fully occluded. Then, two sets of six measurements were obtained, and the mean was calculated. The other eye was tested similarly. All axial length measurements were performed within ± 2 h.

The WAM-550 (Grand Seiko, Hiroshima, Japan) was used to obtain cycloplegic refractive endpoints after full pupil dilation was achieved using 1% cyclopentolate topical ophthalmic solution. This instrument has a resolution of 0.01 D and a range of ± 22 D. Six measurements were obtained for each eye, and the mean was calculated.

### Statistical analysis

The mean of the repeated measurements of both the spherical equivalent (SPHEQ) refractive error and axial length obtained from each eye at each visit were used to determine the two primary outcome measures. The treatment effect at each visit was assessed as the difference between the measurements obtained from the treated (right) eye and that of the control (left) eye. The treatment effects were further adjusted for any between-eye differences observed at the initial (baseline) visit before the initiation of treatment. Finally, the observed *adjusted treatment effects* at each study visit were summed to determine the *cumulative adjusted treatment effect*. This approach considers the small changes observed during each visit and is not biased towards any direction (i.e., positive or negative changes). Furthermore, a positive treatment effect indicated an overall reduction in axial length or reduction in myopic refractive error when comparing the test eye to the control eye.

Given the relatively unknown nature of any possible interocular, interactive effects of the presented stimulus, a trend analysis rather than an individual data-point analysis provides better guidance as to whether or not the treatment has an effect. The cumulative adjusted treatment effect, as opposed to percentage change, was selected, as the latter can result in misleading findings^36,37^. Additionally, this cumulative adjusted change is commonly used to express treatment efficacy^37,38^.

Generalised linear modelling was used to assess the relationship between the cumulative adjusted treatment effect and study visits. The model was constructed to assume a linear relationship with the y-intercept at zero (i.e., no treatment difference at the baseline visit). This methodology allows for the control of the inherent correlation between measurements obtained from the same study subject. Initial analyses were completed to determine the slope of the line relating the study visit and outcome for each individual subject before combining all into a composite measure of effect. A 95% confidence band for the composite regression line was also provided; this differs from a 95% confidence interval, which applies only to a particular study visit.

PASS 2020^39^ software was used for all study power calculations assuming a one-sided hypothesis test (slope > 0), alpha = 0.05, and 80% power. Calculations were performed using effect size (ratio of slope estimate to its variability) since there is no historical data showing the expected slope in this unique study population. With a sample size of 7, the study has 80% power to detect an effect size of 0.94 (large effect). In comparison, the observed effect size estimates for refractive error and axial length are 0.88 and 0.99, respectively. Using the observed estimates, the study powers for refractive error and axial length were 75.6% and 83.2%, respectively.

## Data Availability

The datasets used and/or analysed during the current study available from the corresponding author on reasonable request.
